# Advancements in veterinary medicine: the use of Flowgy for nasal airflow simulation and surgical predictions in big felids (a case study in lions)

**DOI:** 10.3389/fvets.2023.1181036

**Published:** 2024-01-24

**Authors:** Manuel Burgos, Alejandro Pérez-Ramos, Baptiste Mulot, Daniel Sanz-Prieto, Francisco Esteban, Markus Bastir

**Affiliations:** ^1^Dpto. de Ingeniería Térmica y de Fluidos, Universidad Politécnica de Cartagena, Murcia, Spain; ^2^Dpto. de Ecología y Geología, Facultad de Ciencias, Universidad de Málaga, Málaga, Spain; ^3^ZooParc de Beauval and Beauval Nature, Saint-Aignan, France; ^4^Department of Paleobiology, Museo Nacional de Ciencias Naturales, Madrid, Spain; ^5^Servicio Andaluz de Salud, Hospital Universitario Virgen del Rocío, Servicio de Otorrinolaringología, Sevilla, Spain

**Keywords:** airflow, virtual surgery, *Panthera leo*, zoology, wild mammals

## Abstract

Flowgy is a semi-automated tool designed to simulate airflow across the nasal passage and detect airflow alterations in humans. In this study, we tested the use and accuracy of Flowgy in non-human vertebrates, using large felids as the study group. Understanding the dynamics of nasal airflow in large felids such as lions (*Panthera leo*) is crucial for their health and conservation. Therefore, we simulated airflow during inspiration through the nasal passage in three lions (*Panthera leo*), two of which were siblings (specimens ZPB_PL_002 and ZPB_PL_003), without breathing obstructions. However, one of the specimens (ZPB_PL_001) exhibited a slight obstruction in the nasal vestibule, which precluded the specimen from breathing efficiently. Computed tomography (CT) scans of each specimen were obtained to create detailed three-dimensional models of the nasal passage. These models were then imported into Flowgy to simulate the airflow dynamics. Virtual surgery was performed on ZPB_PL_001 to remove the obstruction and re-simulate the airflow. In parallel, we simulated the respiration of the two sibling specimens and performed an obstructive operation followed by an operation to remove the obstruction at the same level and under the same conditions as the original specimen (ZPB_PL_001). Thus, we obtained a pattern of precision for the operation by having two comparable replicas with the obstructed and operated specimens. The simulations revealed consistent airflow patterns in the healthy specimens, demonstrating the accuracy of Flowgy. The originally obstructed specimen and two artificially obstructed specimens showed a significant reduction in airflow through the right nostril, which was restored after virtual surgery. Postoperative simulation indicated an improvement of >100% in respiratory function. Additionally, the temperature and humidity profiles within the nostrils showed marked improvements after surgery. These findings underscore the potential of Flowgy in simulating nasal airflow and predicting the outcomes of surgical interventions in large felids. This could aid in the early detection of respiratory diseases and inform clinical decision-making, contributing to improved veterinary care and conservation efforts. However, further research is needed to validate these findings in other species and explore the potential of integrating Flowgy with other diagnostic and treatment tools in veterinary medicine.

## Introduction

The study of airflow in the nasal passage of felines, particularly large felids such as lions, is a multidisciplinary field of critical importance that intersects veterinary medicine, wildlife conservation, and computational fluid dynamics (1, 2). Nasal airways, a marvel of biological engineering, are complex and intricate structures that play several vital roles in the life of felines, and their importance is often understated (3, 4).

The primary function of the nasal passage is to facilitate respiration. It acts as the first point of contact for air entering the body and is responsible for filtering, warming, and humidifying the inhaled air before it reaches the lungs (5, 6). This process is crucial for maintaining the delicate balance of the respiratory system and ensuring the efficient exchange of gases in the lungs. The nasal passage, which is lined with cilia and mucus, traps dust, allergens, and pathogens, preventing them from reaching the lungs. Any alteration in the structure or function of the nasal airway, such as obstructions or inflammation, can significantly affect the efficiency of this filtration system, leading to difficulties in breathing and increased susceptibility to respiratory infections (7).

In addition to its role in respiration, the nasal cavity houses the olfactory epithelium, which is responsible for the sense of smell (3, 4). The sense of smell is a primary sense in felines that is far more developed than in humans. It plays crucial roles in various aspects of life, including hunting, territory marking, mate selection, and social interaction. Felines rely heavily on their sense of smell to detect prey, recognize territorial boundaries, and communicate with other members of their species. Therefore, any airflow disruption that affects olfactory capabilities can have far-reaching consequences on a feline’s behavior and survival (1).

Moreover, the nasal airways also play a significant role in thermoregulation (5, 6). Large felids such as lions inhabit diverse environments, from the scorching heat of the African savannah to the freezing temperatures of the Siberian taiga. The intricate structure of the nasal passage, with its extensive vascular network, helps dissipate heat and maintain body temperature, which is crucial for survival under extreme conditions. Any alteration in the nasal passage that affects this thermoregulatory function can affect a feline’s ability to adapt to its environment, thereby affecting its survival (1, 5, 6).

These complications can have severe consequences in the wild. Difficulties in breathing can reduce a feline’s hunting efficiency, affecting its ability to catch prey and leading to malnutrition (1, 2). Reduced olfactory capabilities can affect the ability to mark territory, find mates, or avoid predators, thereby increasing vulnerability. Furthermore, the inability to effectively regulate body temperature can affect adaptability to environmental changes, increasing susceptibility to diseases (3, 4).

The lion population is estimated to have declined by approximately 43% over the past two decades, with an estimated 20,000–25,000 lions remaining in the wild (1, 2). The causes of this decline in wild lion populations include a variety of factors, from poaching to climate change and habitat loss. An increase in intensive cattle farming linked to the growing human population and the introduction of domestic animals, such as cats, have led to an increase in bovine pleuropneumonia (8, 9) and feline AIDS virus in lion populations (10, 11).

In addition, the incidence of respiratory disease due to COVID-19 in domestic and farm animals has increased since the 2020 pandemic (12, 13). There has been an increase in cases of respiratory problems in zoo animals in recent years, which are thought to be largely the result of COVID-19 infections (12–14). All these problems generate the need for new actions and vaccination protocols for professionals such as veterinarians and personnel specialized in handling large mammals to control contagions among domestic, farm, and zoo animals (12, 15, 16). Given this situation, human-animal infections and vice versa have increased in recent years. Therefore, a correct respiratory diagnosis, enabling one to distinguish between respiratory problems that are due to the action of COVID-19 and those that are due to other respiratory pathologies, is essential (17, 18). Using Flowgy software as a tool to detect pathologies and conduct virtual surgeries has become indispensable (19–21). In this study, we aimed to demonstrate the potential of Flowgy in large mammals using large felids, such as *Panthera leo*.

Understanding airflow dynamics in the nasal cavities of felines is critical (3, 4). This will provide insights into their physiological adaptations, inform veterinary practices, and contribute to their conservation efforts. By studying the airflow in the nasal cavities, we can better understand how these animals adapt to their environment, communicate, and hunt. Additionally, we can identify and treat respiratory conditions more effectively, thereby improving health and quality of life. Moreover, by understanding how changes in their environment might affect their nasal airflow and, consequently, their ability to regulate body temperature, hunt, and communicate, we can better predict and mitigate the impacts of environmental changes on these animals.

There has been a great demand for non-domestic animals such as wild cats, skunks, possums, and raccoons in recent years (22, 23). These new animals require new protocols for surgical practice and pose a considerable danger to veterinary professionals (24–26).

In the context of veterinary medicine, the application of computational fluid dynamics (CFD) has been a game changer, particularly in the study of respiratory systems in various animals. The respiratory system, with its intricate network of airways and blood vessels, presents a complex fluid flow environment. Understanding airflow dynamics in this system is crucial for diagnosing and treating various respiratory conditions (3–6).

CFD enables non-invasive detailed analysis of airflow patterns within the respiratory system. It can provide valuable data on various parameters, such as pressure, velocity, and turbulence, which can be used to gain insights into the structure–function relationships in the respiratory system. For instance, it can help us to understand how the shape and size of the nasal airways affect airflow and, consequently, the animal’s ability to warm, humidify, and filter inhaled air (3–6).

Moreover, CFD can be used to simulate the effects of certain diseases or conditions that alter the structure of the nasal passage (19–21). Conditions such as polyps, tumors, or congenital deformities can disrupt normal airflow, leading to various complications (7, 17, 18). By simulating these conditions using CFD, veterinarians can better understand the impact on animal respiration and overall health. This can aid in the diagnosis of these conditions and the planning of appropriate treatment strategies.

Furthermore, CFD can be used to predict surgical intervention outcomes. For instance, it can simulate the effects of removing a polyp or tumor or correcting a congenital deformity on the airflow in the nasal passage (7–11). This can provide valuable information for clinical decision-making, helping veterinarians predict the likely outcomes of surgery and plan the best course of action.

In conclusion, applying CFD in veterinary medicine represents a significant advancement in this field. It is a powerful tool for understanding the complex dynamics of airflow in the respiratory system, aiding in diagnosing and treating various conditions. As technology advances, its application in veterinary medicine will likely expand, opening new avenues for research and clinical practice.

Flowgy software was selected to perform all preprocessing, airflow simulations, and post-processing.^1^ It is an innovative semi-automated tool designed to simulate and analyze airflow across nasal passages (19–21). Its primary function is to apply highly accurate CFD analysis to models in a short time. Over the last few years, the analytical capabilities of Flowgy have been improved at different levels, from generating more accurate virtual models to the analytical power of CFD and adding the capability of virtual surgeries (27–32). The last aspect was to focus on this study, which used large carnivores to demonstrate the versatility of Flowgy in the fields of veterinary medicine and zoology.

The main goals of this study were to test (i) virtual surgery tools by detecting perturbations in healthy cases (small intentional obstructions), (ii) the computational limits of Flowgy using large mammals with complex nasopharyngeal topology, and (iii) the results of the analysis using hyperbolic curves and compare them with the standard curves of efficient and inefficient hyperbolic functions.

## Materials and methods

The study material consisted of three *Panthera leo krugeri* specimens, of which two were siblings (ZPB_PL_002 and ZPB_PL_003), and the other specimen (ZPB_PL_001) was a subadult individual. They were captive specimens from the ZooParc de Beauval and Beauval Nature. The specimens were anesthetized for a tomographic study for a veterinary protocol review. One of the measures of the protocol is to carry out medical studies (medical checkups) to detect possible pathologies and to conduct routine follow-ups on the health of the animals. The acronyms used to register the study specimens were based on the provenance, i.e., Zooparc de Beauval (ZPB), and the species (PL, *Panthera leo*).

### Data acquisition

The study material was tomographed at the Beauval Veterinary Institute. The acquisition parameters were 120 kV and 499 mA. The data obtained from the three specimens at the cranial level were in 16-bit format with a 512 × 512 pixel size. The reconstruction of the images of each specimen gave the following voxel sizes with their specific image numbers: ZPB_PL_001 had 460 slices with a slice thickness of 0.6 mm and a voxel size of 0.375 mm (X, Y) and 0.600 mm (Z); ZPB_PL_002 had 591 slices with a slice thickness of 0.6 mm and a voxel size of 0.527 mm (X, Y) and 0.600 mm (Z); and ZPB_PL_003 had 454 slices with a slice thickness of 0.8 mm and a voxel size of 0.516 mm (X, Y) and 0.804 mm (Z).

### Computational CFD workflow

#### Pre-processing

**Figure 1:**
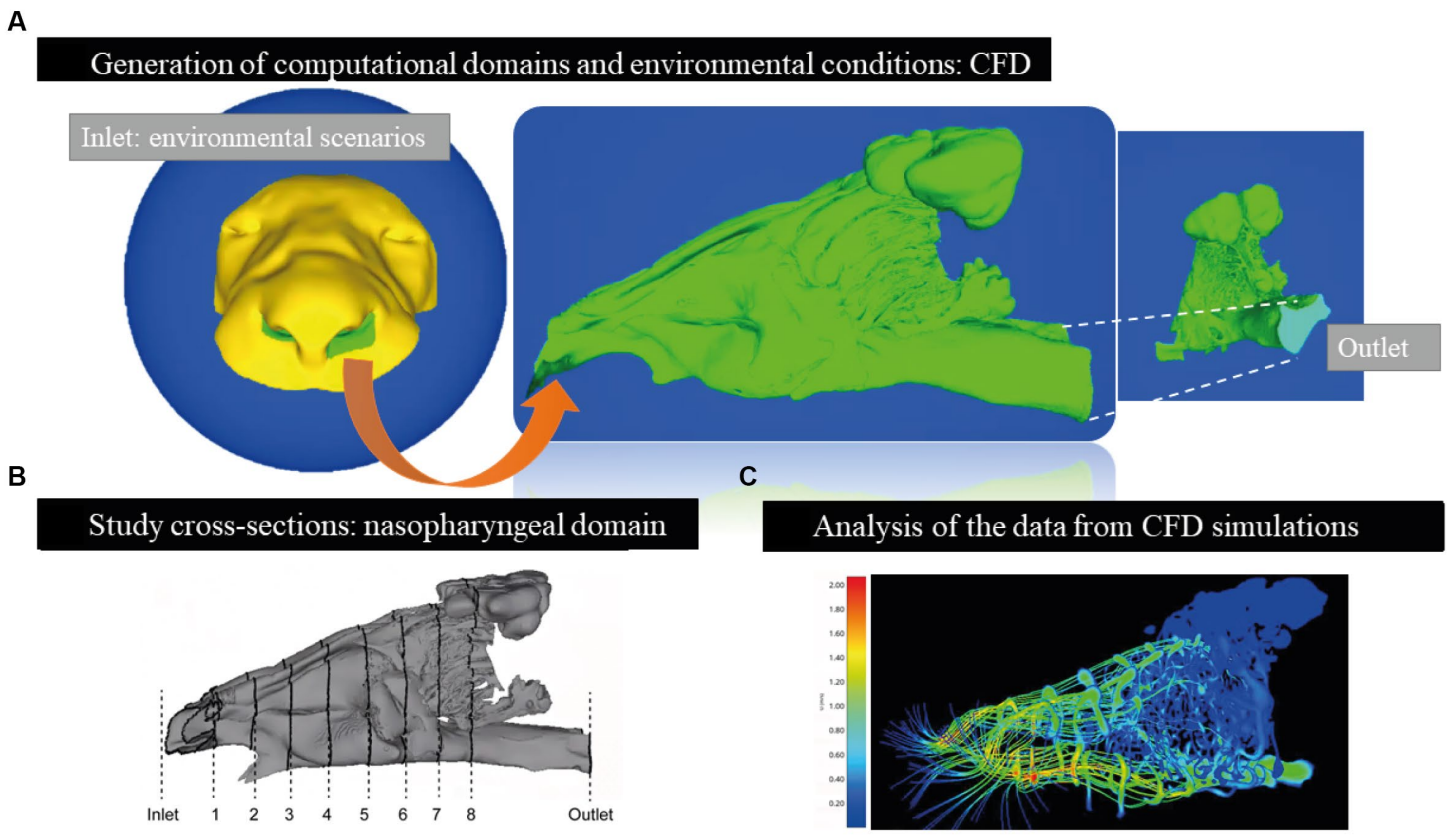
Virtual surgical process in *Panthera leo* specimens. **(A)** Import of tomographic data and generation of computational domains from the produced meshes. Input (in blue) in a spherical shape represents the external boundary conditions for simulating environmental scenarios. Surface domain 1 (in yellow) represents the external topology of the model where the air will interact when entering through the nostrils. Domain 2 (study of the nasopharyngeal tract; in green) represents where the airflow will be simulated. Domain 3 (output; in light blue) represents the system’s simulated outflow. At this point, the computational fluid dynamics (CFD) analysis was conducted. **(B)** Study planes cut along the nasopharyngeal tract for the subsequent analysis of the simulated flow. The first cut (inlet) is the air inlet (representing the atmosphere) and is generated in the cutting plane closest to the nostrils from the outside, and the last cut plane (outlet) is the air outlet from the nasopharyngeal tract. The remaining eight cuts are generated anatomically in a homologous manner, with the first one (cut 1) behind the nostrils and cut 8 in the proximal position of the choana. The remaining six cuts are added equidistantly. **(C)** Quantification of pressure, temperature, and humidity variables in the air simulated by CFD. In this example, airflow speed maps or streamline lines (m/s) are applied, where blue represents low speeds or stationary air, and red represents high speeds.

We used Flowgy software (see text footnote 1) for virtual morphological three-dimensional image preparation, semi-automatic segmentation of the CT dataset, and quantitative simulations of the physical properties of nasal airflow dynamics (27, 28) (see Figure 1).

**Table 1:** Data on *Panthera leo* specimen ZPB_PL_001.

Study slices	Distance	Temp. 28	Temp. 40	Temp. 5	Average temp.	Humid. 60	Humid. 05	Humid. 10	Average H.
1	0.0000	−1.0000	−1.0000	−1.0000	−1.0000	−1.0000	−1.0000	−1.0000	−1.0000
2	0.1806	−0.5035	−0.5027	−0.5058	−0.5040	−0.5143	−0.5159	−0.5166	−0.5156
3	0.2977	−0.0961	−0.0953	−0.0978	−0.0964	−0.0988	−0.1012	−0.1032	−0.1011
4	0.4147	−0.0193	−0.0193	−0.0196	−0.0194	−0.0137	−0.0169	−0.0177	−0.0161
5	0.5318	−0.0017	−0.0020	−0.0017	−0.0018	0.0085	0.0049	0.0047	0.0061
6	0.6488	−0.0002	0.0000	−0.0001	−0.0001	0.0108	0.0074	0.0069	0.0084
7	0.7659	0.0000	0.0000	0.0000	0.0000	0.0111	0.0076	0.0073	0.0087
8	0.8829	0.0000	0.0000	−0.0001	0.0000	0.0111	0.0074	0.0071	0.0085
9	1.0000	−0.0133	−0.0133	−0.0129	−0.0132	−0.0026	−0.0061	−0.0063	−0.0050

Standardization of temperature and humidity data obtained from the three simulations (28°C at 60% RH, 40°C at 5% RH, and 5°C at 10% RH) applying the method described by Burgos (32).

The data obtained were imported into Flowgy. The first step was the isometric conversion of the voxels to a size of 0.4 (29). This method of resampling using a binary process is essential for generating virtual models for CFD analyses.

The CT scans were segmented to create three-dimensional models of the face (yellow), nasal airways, paranasal sinuses, nasopharynx (green), and spherical external region (blue) to simulate the natural inflow of air during inspiration (inlet). The final section of the nasopharyngeal passage represents the outflow domain of the inspired flow (light blue) and the outlet. The details of the computational domains are presented in Figure 1A. Thus, we used a non-truncated computational domain, which consists of all or part of the subject’s head, surrounded by an atmosphere from which air is inhaled through the nasal passage (27, 28, 30, 31) (see Figure 1A).

**Table 2:** Data on the operated *Panthera leo* specimen ZPB_PL_001.

Study slices	Distance	Temp. 28	Temp. 40	Temp. 5	Average temp.	Humid. 60	Humid. 05	Humid. 10	Average H.
1	0.0000	−1.0000	−1.0000	−1.0000	−1.0000	−1.0000	−1.0000	−1.0000	−1.0000
2	0.1806	−0.4338	−0.4327	−0.4356	−0.4340	−0.4431	−0.4453	−0.4460	−0.4448
3	0.2977	−0.0669	−0.0660	−0.0681	−0.0670	−0.0662	−0.0693	−0.0708	−0.0688
4	0.4147	−0.0122	−0.0120	−0.0125	−0.0122	−0.0042	−0.0076	−0.0082	−0.0067
5	0.5318	−0.0011	−0.0013	−0.0011	−0.0012	0.0101	0.0065	0.0063	0.0076
6	0.6488	−0.0001	0.0000	−0.0001	−0.0001	0.0117	0.0081	0.0078	0.0092
7	0.7659	0.0000	0.0000	0.0000	0.0000	0.0121	0.0083	0.0080	0.0095
8	0.8829	0.0000	0.0000	0.0000	0.0000	0.0121	0.0083	0.0080	0.0095
9	1.0000	−0.0151	−0.0153	−0.0147	−0.0151	−0.0036	−0.0072	−0.0076	−0.0061

Standardization of temperature and humidity data obtained from the three simulations (28°C at 60% RH, 40°C at 5% RH, and 5°C at 10% RH) applying the method described by Burgos (32).

**Table 3:** Data on *Panthera leo* specimen ZPB_PL_003.

Study slices	Distance	Temp. 28	Temp. 40	Temp. 5	Average temp.	Humid. 60	Humid. 05	Humid. 10	Average H.
1	0.0000	−1.0000	−1.0000	−1.0000	−1.0000	−1.0000	−1.0000	−1.0000	−1.0000
2	0.2143	−0.2496	−0.2470	−0.2535	−0.2501	−0.2595	−0.2610	−0.2645	−0.2616
3	0.3265	−0.0785	−0.0772	−0.0804	−0.0787	−0.0802	−0.0828	−0.0844	−0.0825
4	0.4388	−0.0200	−0.0200	−0.0205	−0.0202	−0.0140	−0.0171	−0.0179	−0.0163
5	0.5510	−0.0049	−0.0047	−0.0047	−0.0048	0.0059	0.0025	0.0022	0.0035
6	0.6633	−0.0031	−0.0033	−0.0030	−0.0032	0.0075	0.0043	0.0039	0.0052
7	0.7755	−0.0034	−0.0033	−0.0033	−0.0034	0.0075	0.0043	0.0039	0.0052
8	0.8878	−0.0066	−0.0067	−0.0064	−0.0065	0.0049	0.0016	0.0011	0.0025
9	1.0000	−0.0171	−0.0173	−0.0166	−0.0170	−0.0049	−0.0083	−0.0086	−0.0073

Standardization of temperature and humidity data obtained from the three simulations (28°C at 60% RH, 40°C at 5% RH, and 5°C at 10% RH) applying the method described by Burgos (32).

### Experimental and boundary conditions

#### Virtual surgery

**Figure 2:**
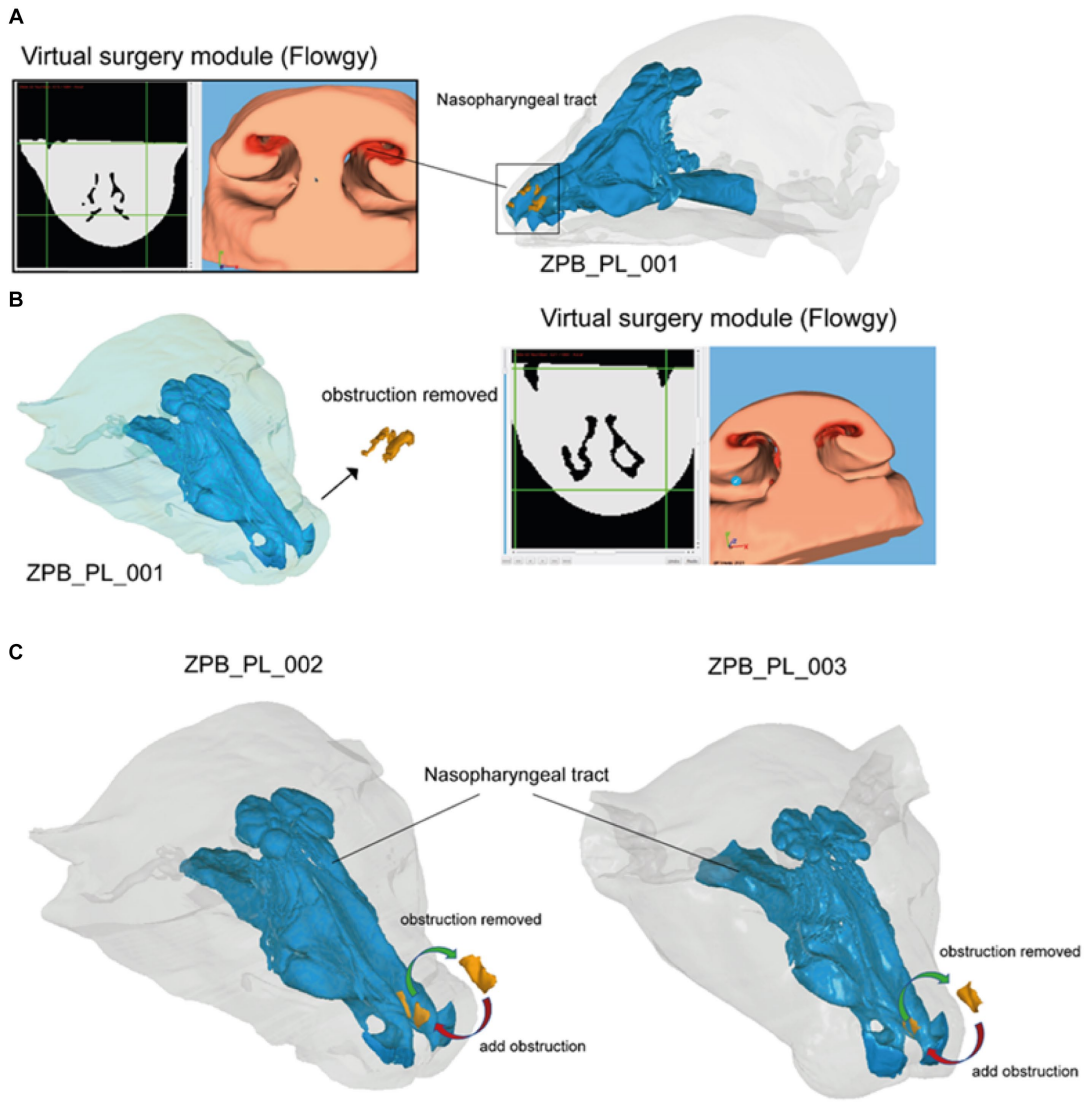
The process of using the virtual surgery module of the Flowgy program. **(A)** Case 01 (ZPB_PL_001): exploration of the frontal nasal meatus obstruction using the Flowgy software module tools. **(B)** Operation using the virtual surgery module tools to remove the obstruction. This resulted in Case 01 after the operation and without the obstruction. **(C)** Obstructive operation of Cases 02 (ZPB-PL_002) and 03 (ZPB-PL_003) by adding material in the frontal nasal meatuses using a virtual scalpel and virtual surgery module polylines. In this step, the red arrows indicate the process. The second step is to reoperate on Cases 02 and 03 by means of a de-obstructive surgery using the same Flowgy module tools, but in this process, removing, instead of adding, material (green arrows).

The advent of virtual surgery software, particularly Flowgy, has offered unprecedented possibilities for veterinary surgical planning. In this report, three cases were examined (ZPB_PL_001 [Case 01], ZPB_PL_002 [Case 02], and ZPB_PL_003 [Case 03]), each requiring distinct surgical interventions in the nasal area. This study aimed to provide a comprehensive overview of how virtual surgery can aid in unblocking and intentionally obstructing specific nasal passages for various medical necessities (Figure 2).

**Table 4:** Data on the obstructed *Panthera leo* specimen ZPB_PL_003.

Study slices	Distance	Temp. 28	Temp. 40	Temp. 5	Average temp.	Humid. 60	Humid. 05	Humid. 10	Average H.
1	0.0000	−1.0000	−1.0000	−1.0000	−1.0000	−1.0000	−1.0000	−1.0000	−1.0000
2	0.2143	−0.6733	−0.6718	−0.6738	−0.6730	−0.6802	−0.6822	−0.6809	−0.6811
3	0.3265	−0.1785	−0.1764	−0.1818	−0.1789	−0.1842	−0.1863	−0.1895	−0.1867
4	0.4388	−0.0432	−0.0426	−0.0450	−0.0436	−0.0401	−0.0430	−0.0451	−0.0427
5	0.5510	−0.0056	−0.0053	−0.0056	−0.0055	0.0055	0.0020	0.0015	0.0030
6	0.6633	−0.0032	−0.0033	−0.0031	−0.0032	0.0085	0.0047	0.0043	0.0058
7	0.7755	−0.0034	−0.0033	−0.0033	−0.0034	0.0085	0.0049	0.0045	0.0060
8	0.8878	−0.0066	−0.0067	−0.0064	−0.0065	0.0059	0.0022	0.0017	0.0033
9	1.0000	−0.0171	−0.0173	−0.0166	−0.0170	−0.0039	−0.0076	−0.0080	−0.0065

Standardization of temperature and humidity data obtained from the three simulations (28°C at 60% RH, 40°C at 5% RH, and 5°C at 10% RH) applying the method described by Burgos (32).

##### Case 01: unblocking nasal passages (ZPB_PL_001)

In Case 01, the focus was on eliminating an obstruction in the nasal vestibule (3, 4). The module used for this operation was replete with a set of virtual tools that mimicked the real-world surgical environment (Figure 2A). A notable tool is the virtual scalpel, which facilitates the removal of material from a three-dimensional model representing the anatomical structure of the subject animal. The scalpel was calibrated to ensure precision in the incisions and to minimize the removal of non-target tissues (27, 28, 30, 31). In addition, the module incorporates polyline technology, which enables the user to manipulate partitions or augment materials in a manner that adheres to the septum and meatus anatomical patterns. In the case of ZPB_PL_001, the obstruction was successfully excised by carefully maneuvering the virtual scalpel tool along a planned polyline path, as shown in Figure 2B. The result showed a clear frontal nasal meatus, making it a suitable candidate for subsequent simulations analyzing fluid dynamics and other variables of interest (Tables 1–8).

**Table 5:** Data on the operated *Panthera leo* specimen ZPB_PL_003.

Study slices	Distance	Temp. 28	Temp. 40	Temp. 5	Average temp.	Humid. 60	Humid. 05	Humid. 10	Average H.
1	0.0000	−1.0000	−1.0000	−1.0000	−1.0000	−1.0000	−1.0000	−1.0000	−1.0000
2	0.2143	−0.3153	−0.3129	−0.3189	−0.3157	−0.3250	−0.3267	−0.3299	−0.3272
3	0.3265	−0.0890	−0.0879	−0.0910	−0.0893	−0.0903	−0.0934	−0.0950	−0.0929
4	0.4388	−0.0224	−0.0220	−0.0230	−0.0225	−0.0160	−0.0193	−0.0203	−0.0185
5	0.5510	−0.0049	−0.0047	−0.0048	−0.0048	0.0068	0.0029	0.0026	0.0041
6	0.6633	−0.0034	−0.0033	−0.0033	−0.0034	0.0081	0.0045	0.0043	0.0057
7	0.7755	−0.0036	−0.0033	−0.0035	−0.0035	0.0085	0.0047	0.0043	0.0058
8	0.8878	−0.0067	−0.0067	−0.0065	−0.0066	0.0059	0.0020	0.0017	0.0032
9	1.0000	−0.0174	−0.0180	−0.0170	−0.0175	−0.0042	−0.0079	−0.0084	−0.0068

Standardization of temperature and humidity data obtained from the three simulations (28°C at 60% RH, 40°C at 5% RH, and 5°C at 10% RH) applying the method described by Burgos (32).

**Table 6:** Data on *Panthera leo* specimen ZPB_PL_002.

Study slices	Distance	Temp. 28	Temp. 40	Temp. 5	Average temp.	Humid. 60	Humid. 05	Humid. 10	Average H.
1	0.0000	−1.0000	−1.0000	−1.0000	−1.0000	−1.0000	−1.0000	−1.0000	−1.0000
2	0.2143	−0.2650	−0.2633	−0.2687	−0.2657	−0.2764	−0.2783	−0.2804	−0.2784
3	0.3265	−0.0736	−0.0727	−0.0755	−0.0739	−0.0740	−0.0767	−0.0784	−0.0764
4	0.4388	−0.0173	−0.0173	−0.0179	−0.0175	−0.0111	−0.0142	−0.0149	−0.0134
5	0.5510	−0.0031	−0.0033	−0.0031	−0.0032	0.0075	0.0043	0.0039	0.0052
6	0.6633	−0.0133	−0.0133	−0.0129	−0.0132	−0.0026	−0.0061	−0.0063	−0.0050
7	0.7755	−0.0008	−0.0007	−0.0007	−0.0007	0.0101	0.0067	0.0065	0.0078
8	0.8878	−0.0047	−0.0047	−0.0045	−0.0046	0.0068	0.0036	0.0032	0.0046
9	1.0000	−0.0205	−0.0207	−0.0198	−0.0203	−0.0081	−0.0115	−0.0119	−0.0105

Standardization of temperature and humidity data obtained from the three simulations (28°C at 60% RH, 40°C at 5% RH, and 5°C at 10% RH) applying the method described by Burgos (32).

##### Cases 02 and 03: inducing controlled obstructions (ZPB_PL_002 and ZPB_PL_003)

In contrast to Case 01, Cases 02 and 03 required the intentional obstruction of specific nasal regions (Figure 1C). This is often required for many reasons, including, but not limited to, experimental trials on obstructive respiratory conditions. In these cases, a complementary set of tools was used, prominently featuring contour master polylines and an alternate setting on the virtual scalpel to add rather than remove material (28, 31). This procedure was methodological. Contour master polylines were initially used to delineate the region where the obstruction was introduced. These polylines function as dynamic guides for the virtual scalpel, which adds material to the three-dimensional model in this particular mode. The integrity of the three-dimensional model was maintained throughout, ensuring that the added material coherently followed the topological and anatomical patterns of the existing structures. The operations for Cases 02 and 03 are illustrated in Figure 2C. The objective was to perform an induced obstructive operation with the same characteristics (Cases 02 and 03) and at the same location within the nasal tract as the diseased specimen (Case 01), scaling the obstruction (orange object in Figure 2) to the proportional size of the healthy specimens (Figure 1C; Table 9).

**Table 7:** Data on the obstructed *Panthera leo* specimen ZPB_PL_002.

Study slices	Distance	Temp. 28	Temp. 40	Temp. 5	Average temp.	Humid. 60	Humid. 05	Humid. 10	Average H.
1	0.0000	−1.0000	−1.0000	−1.0000	−1.0000	−1.0000	−1.0000	−1.0000	−1.0000
2	0.2143	−0.6881	−0.6887	−0.6890	−0.6886	−0.2751	−0.6995	−0.6969	−0.5572
3	0.3265	−0.1763	−0.1720	−0.1770	−0.1751	−0.0759	−0.1802	−0.1835	−0.1466
4	0.4388	−0.0409	−0.0400	−0.0424	−0.0411	−0.0104	−0.0400	−0.0421	−0.0309
5	0.5510	−0.0038	−0.0040	−0.0040	−0.0039	0.0088	0.0038	0.0032	0.0053
6	0.6633	−0.0141	−0.0133	−0.0130	−0.0135	−0.0023	−0.0056	−0.0058	−0.0046
7	0.7755	−0.0008	−0.0007	−0.0007	−0.0007	0.0111	0.0074	0.0071	0.0085
8	0.8878	−0.0047	−0.0047	−0.0045	−0.0046	0.0078	0.0043	0.0039	0.0053
9	1.0000	−0.0210	−0.0207	−0.0198	−0.0205	−0.0075	−0.0108	−0.0112	−0.0098

Standardization of temperature and humidity data obtained from the three simulations (28°C at 60% RH, 40°C at 5% RH, and 5°C at 10% RH) applying the method described by Burgos (32).

**Table 8:** Data on the operated *Panthera leo* specimen ZPB_PL_002.

Study slices	Distance	Temp. 28	Temp. 40	Temp. 5	Average temp.	Humid. 60	Humid. 05	Humid. 10	Average H.
1	0.0000	−1.0000	−1.0000	−1.0000	−1.0000	−1.0000	−1.0000	−1.0000	−1.0000
2	0.2143	−0.3307	−0.3293	−0.3341	−0.3314	−0.3419	−0.3440	−0.3459	−0.3439
3	0.3265	−0.0841	−0.0833	−0.0861	−0.0845	−0.0841	−0.0873	−0.0889	−0.0868
4	0.4388	−0.0197	−0.0193	−0.0204	−0.0198	−0.0130	−0.0164	−0.0173	−0.0156
5	0.5510	−0.0031	−0.0033	−0.0031	−0.0032	0.0085	0.0047	0.0043	0.0058
6	0.6633	−0.0136	−0.0133	−0.0132	−0.0134	−0.0020	−0.0058	−0.0058	−0.0045
7	0.7755	−0.0010	−0.0007	−0.0009	−0.0008	0.0111	0.0072	0.0069	0.0084
8	0.8878	−0.0048	−0.0047	−0.0046	−0.0047	0.0078	0.0040	0.0039	0.0053
9	1.0000	−0.0208	−0.0213	−0.0202	−0.0208	−0.0075	−0.0110	−0.0117	−0.0101

Standardization of temperature and humidity data obtained from the three simulations (28°C at 60% RH, 40°C at 5% RH, and 5°C at 10% RH) applying the method described by Burgos (2014) (32).

**Table 9:** Data on the obstructive material extracted from each specimen and nasopharyngeal length measurements of each specimen.

Specimens	ZPB_PL_001	ZPB_PL_002	ZPB_PL_003
Length (nostrils-choana) mm	80.28	155.57	157.24
Material obstructed	ZPB_PL_001	ZPB_PL_002	ZPB_PL_003
Length mm	18.95	26.66	21.23
Width mm	6.98	19.4	19.21
Surface mm^2^	336.49	763.85	610.92
Volume mm^3^	695.55	984,11	856.89

Data on the size of the nasopharyngeal tract (nasal entrance or nostril to the exit or choana). Measurements of the material extracted in specimen ZPB_PL_001 as well as those added in the replicate specimens (ZPB_PL_002 and ZPB_PL_003) to simulate the obstruction.

Specimens ZPB_PL_003 and ZPB_PL_002 were twin brothers; therefore, they were ideal specimens for a replicate study. Based on this genetic approach, we performed a mock operation on these two specimens and validated the success of the operation and its accuracy. In these cases, virtual obstructive operations were performed in the same nasal region where the obstruction was detected in specimen ZPB_PL_001. The next phase was the operation of such an obstruction so that the two specimens (siblings) could return to their initial state of respiratory function. The results are shown in Figure 2C. The data for the materials extracted from the different specimens were similar. It must be considered that specimen ZPB_PL_001 is a subadult; therefore, the extracted material that caused the obstruction was smaller than that in the replicate study. However, the same conditions were used based on the location and form of the obstruction. The results are summarized in Table 9.

In summary, unblocking and controlled blocking surgeries were executed using the virtual surgery module of the Flowgy software. The results demonstrated high levels of accuracy and feasibility for both intervention types. Importantly, these simulations will pave the way for future surgical plans and additional simulations to improve patient outcomes.

#### Fluid dynamics simulation

To simulate inspiration, the input to the computational domain was outside the surroundings of the subject’s head, where the value of a known atmospheric pressure (101,325 Pa) was set. We modeled the efficiency of the nasal airflow in three different environmental scenarios: a temperate forest region (28°C, 60% RH), a warm desert region (40°C, 5% RH), and a cold or high mountain region (5°C, 10% RH). (5). Boundary conditions within the nasal passage, i.e., the area between the internal part of the nostrils and the nasopharynx, were defined and set to 100% relative humidity and 311.65 K (38.5°C) (5, 6) (Figure 1A). The Navier–Stokes equations were solved in a steady state with laminar and compressible flows using the open-source CFD software Open FOAM.^2^ An appropriate pressure drop was applied between the atmosphere and the nasopharynx in each case to obtain an approximate flow rate of 15 L/min (31, 32) (see Figure 1A).

We generated unstructured tetrahedral meshes with sizes ranging from 7 to 30 million tetrahedral cells to solve the governing equations of the fluid flow and performed a mesh convergence study to evaluate the accuracy of the values in the simulations (see Table 10) (27, 28, 30–32). The numerical solutions of the equations included temperature (provided in Kelvin units but converted to degrees Celsius for a correct understanding of the study), velocity (m/s), and absolute humidity (Kg/m^3^). Specifically, we estimated absolute and standardized (relative) values of temperature, humidity, and velocity and compared them at specific sections between the external environment and the choanae, represented by different functional regions of the entire nasal airway (Figure 1A). These sections or cuts were equidistant and homologous in all three specimens. Eight anatomical sections and the inlet section represented the atmosphere and the outlet section of the nasopharyngeal tract (Figure 1B) (27, 28, 30–32).

**Table 10:** A mesh convergence test was performed using the specimen from Case 01 (similar to Burgos, (32)).

Mesh size	Volumetric elements (approx.)	Nr V (m/s)	RE (%)	Nr P (Pa)	RE (%)	Nl V (m/s)	RE (%)	Nl (Pa)	RE (%)	Ch V (m/s)	RE (%)	Ch P (Pa)	RE (%)
Coarse	1.5 million	0.305	4.98	101,312	0.16	0.735	0.97	101,311	0.11	0.270	2.31	101,302	0.02
Medium	3.5 million	0.317	1.24	101,311	0.08	0.740	0.30	101,310	0.04	0.275	0.51	101,301	0.01
Finest	7.0 million	0.321	–	101,310	–	0.742	–	101,309	–	0.276	–	101299.992	–

Three mesh resolutions were applied in this specimen: coarse (approximately 1.5 million elements), medium (approximately 3.5 million elements), and fine (approximately 7 million elements, approximately half of that used in this study). Pressure (Pa) and velocity (m/s) measurements were obtained at common locations (right and left nostrils and choana) and compared between mesh sizes. Relative error (RE) as a percentage, velocity (V), pressure (P), nostril right (Nr), nostril left (Nl), Choana (Ch).

## Results

**Figure 3:**
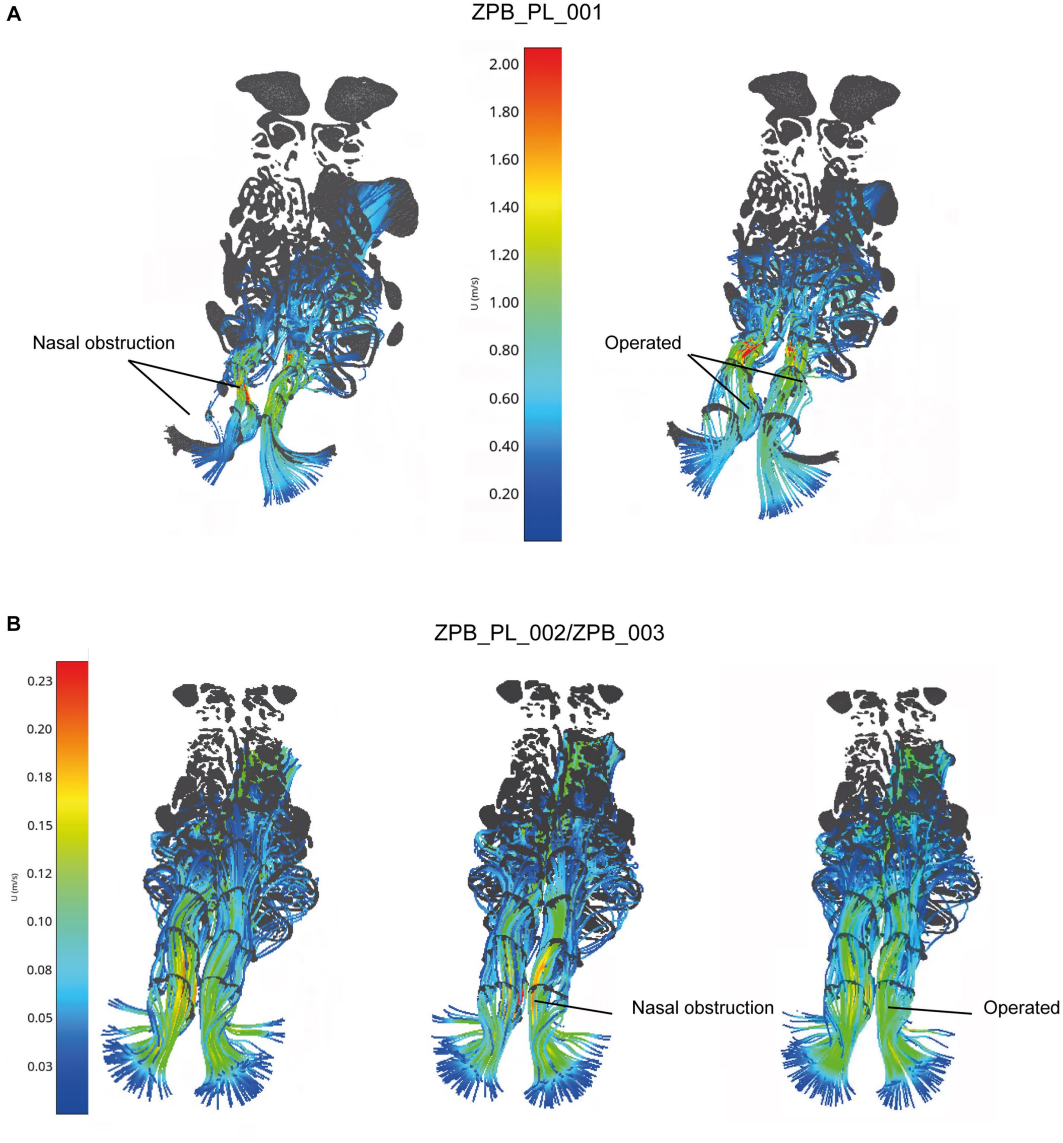
CFD simulations for the three specimens (Case 01 [ZPB_PL_001], Case 02 [ZPB_PL_002], and Case 03 [ZPB_PL_003]). The airflow lines (streamlines) are represented in all cases with a color pattern based on the velocity values (m/s), i.e., low velocities represented using cold colors and high velocities represented using warm colors. Nasal obstruction is indicated, where airflow is minimal or velocities are high due to reduced air passage. Operated nasal passages increase the airflow through a larger opening of the nasal passages, returning the airflow velocities to normal values.

**Figure 4:**
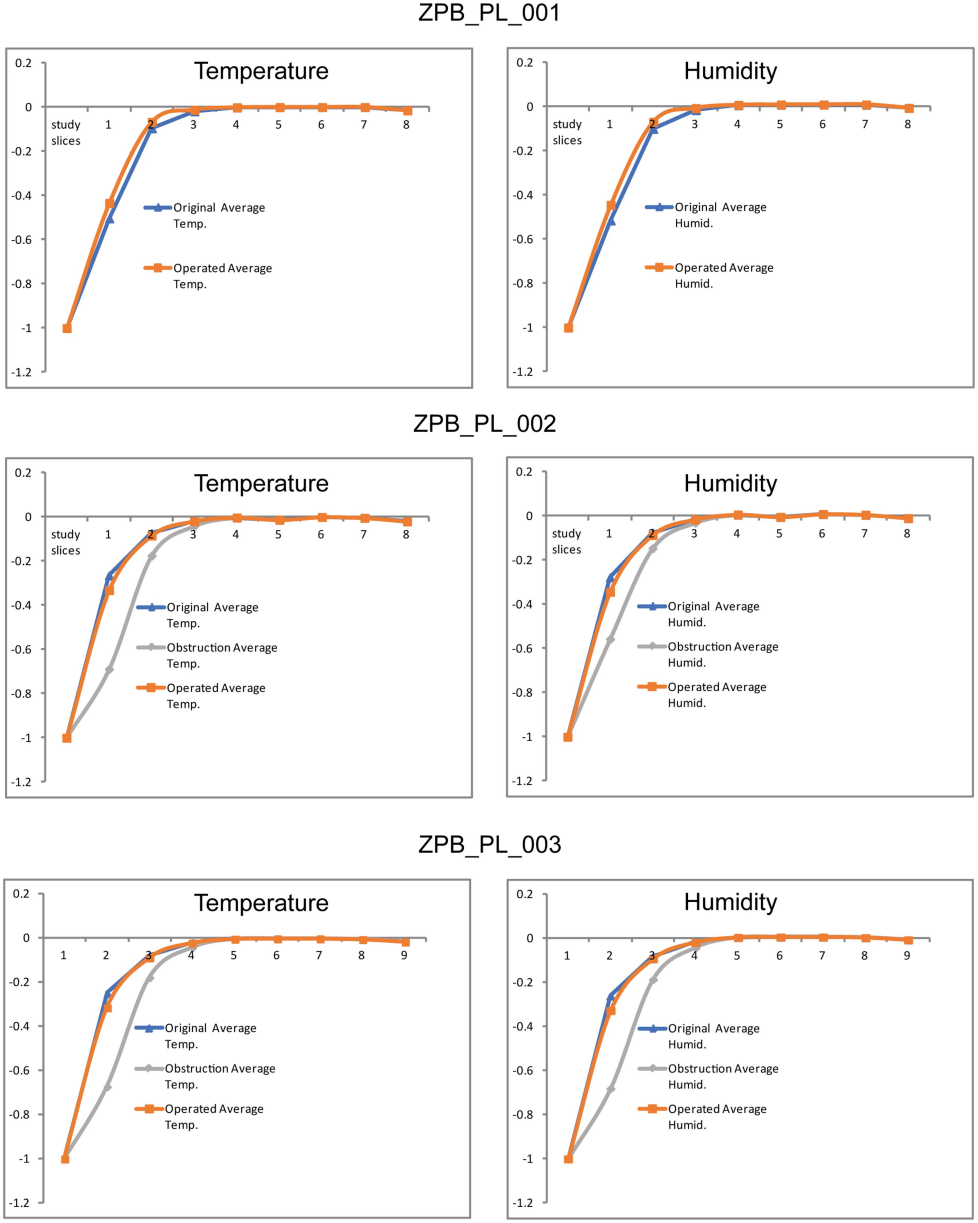
Plots of the results obtained from the CFD simulations in the three scenarios (28°C at 60% RH, 40°C at 5% RH, and 5°C at 10% RH) from the standardized data in Tables 1–8, following the study by Burgos, 2014 (32). Case 01 (ZPB_PL_001), Case 02 (ZPB_PL_002), and Case 03 (ZPB_PL_003) are shown.

The results supported the reliability and accuracy of the operation using large felids, such as *Panthera leo*, as a case study (Figures 1C, 2). As shown in Figure 3A, the obstruction in ZPB_PL_001 (Case 01) was very high, reducing the inhalation of air through the right nostril. Once operated on, the flow through the right nostril was restored. Regarding the replicate lions (Figure 3B), the comparison between the initial situation, before the obstructive surgery, and the final operating situation showed that the flow was the same, both in topology and velocity. With regard to the obstructive situation, an increase in velocity at the level of the obstruction was observed. This was because the same volume of flow must pass through a narrower channel, which generates regions with high velocities. This indicated the high accuracy of the Flowgy software in detecting small obstructions in detail. With respect to the data obtained, the temperature and humidity graphs in Figure 4 show that the data for the operated situation (orange) improved from those of the initial situation (blue). The data for Cases 02 and 03 in Figure 4 indicate that the obstruction (gray) generated a greater negative response in the respiratory function. This is because the two specimens used were two adult lions with a larger allometry and size than *Panthera leo* Case 01. Nevertheless, the operation on the two lions (orange) resulted in the return of respiratory function very close to or equal to the initial situation (blue). We followed the principle of similarity in the resolution of the flow during the acclimation process, detailed by Burgos (32), to observe changes in the acclimation function based on the temperature and absolute humidity of each specimen. This equation is expressed as follows:


$$\begin{array}{l} T\;\left(\mathrm{average}\;\mathrm{of}\;\mathrm{each}\;\mathrm{cut}\right)-T\;\left(\mathrm{internal}\;\mathrm{body}\right)/\\ T\;\left(\mathrm{internal}\;\mathrm{body}\right)-T\;\left(\mathrm{external}\right)\text{.} \end{array}$$

Each slice from the standardized and homologous coordinates was divided by the final slice to convert the distance into values from 0 to 1 as a distance index to represent the standardized temperature and humidity data based on the expressed formula. Following the nomenclature of Burgos et al. (32), the average temperature and absolute humidity data were collapsed into a hyperbolic function. The x-axis represents the dimensionless distance d* = d/dc, where dc is the distance from the nares to the choana. The y-axis represents dimensionless temperature and humidity:


$$\theta =\left(T-Tw\right)/\left(Tw-To\right)\text{,}$$

where T is the average temperature at each study point, Tw is the average nasopharyngeal wall temperature, and To is the average outlet temperature.


$$\theta =\left(H-Hw\right)/\left(Hw-Ho\right)\text{,}$$

where H is the average humidity at each study point, Hw is the average nasopharyngeal wall humidity, and Ho is the average outlet humidity.

Subsequently, the data were fitted to a hyperbolic function by means of the differences in the sum of the squares with two values that acted as operators (α and β), as in the study by Burgos (2014) (32). This formula was used so that the collapsed average data matched the minimum square of the data to the function of the hyperbolic curve:

Hyperbolical formula:


$$\mathrm{TANH}\left(\frac{d^{*}+\alpha }{\beta }-1\right)\text{,}$$

**Figure 5:**
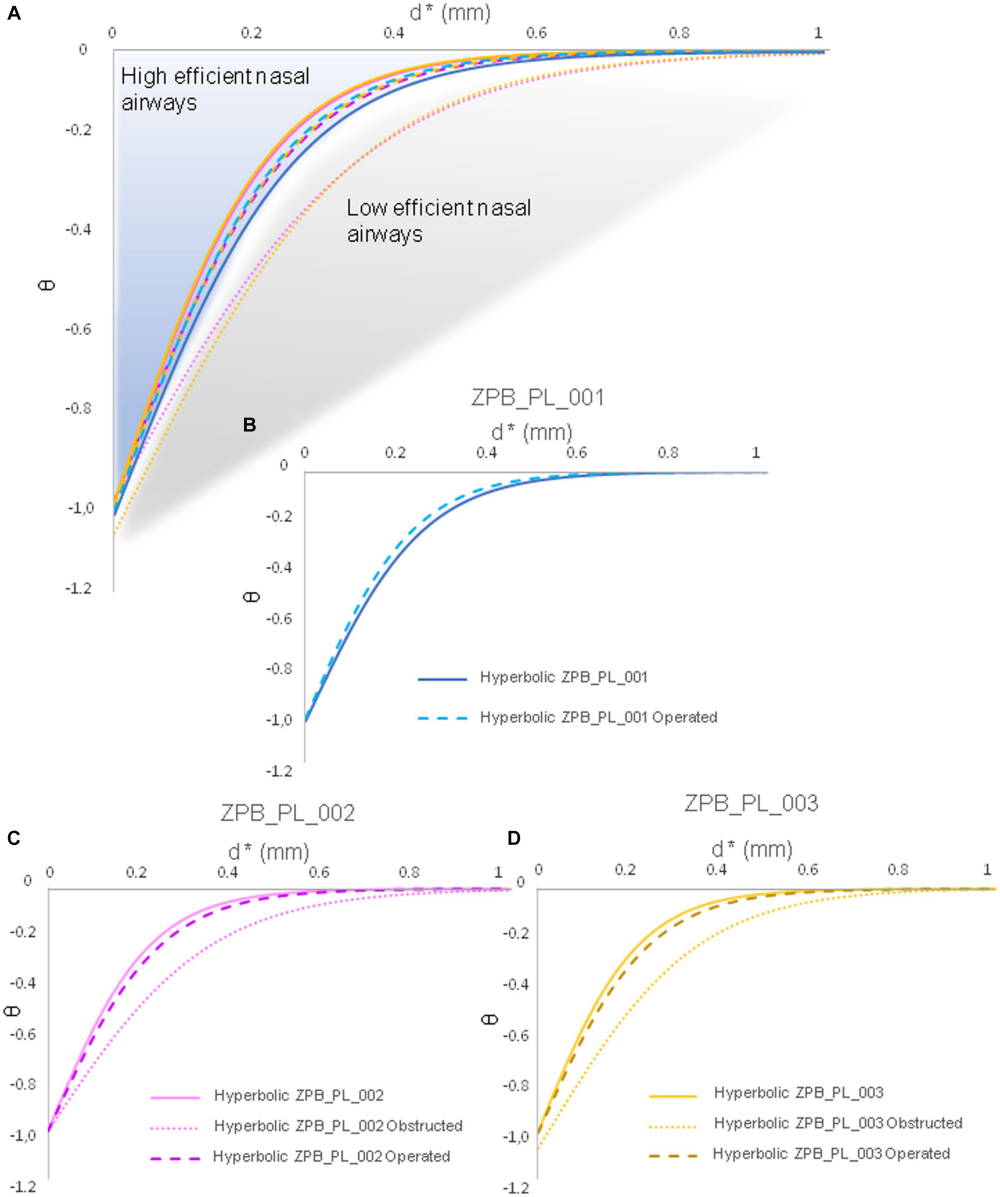
Temperature data collapsed into a hyperbolic function. These hyperbolic functions are generated based on the functions used by Burgos, 2014 (32). The average temperature data are from Tables 1–8. These hyperbolic functions describe the acclimation function specific to each nasal airway of each specimen. **(A)** General cases of all specimens. **(B)** Functional hyperbola of specimen ZPB_PL_001. **(C)** Functional hyperbola of specimen ZPB_PL_002. **(D)** Functional hyperbola of specimen ZPB_PL_003. Case 01 (ZPB_PL_001), Case 02 (ZPB_PL_002), and Case 03 (ZPB_PL_003) are shown.

**Figure 6:**
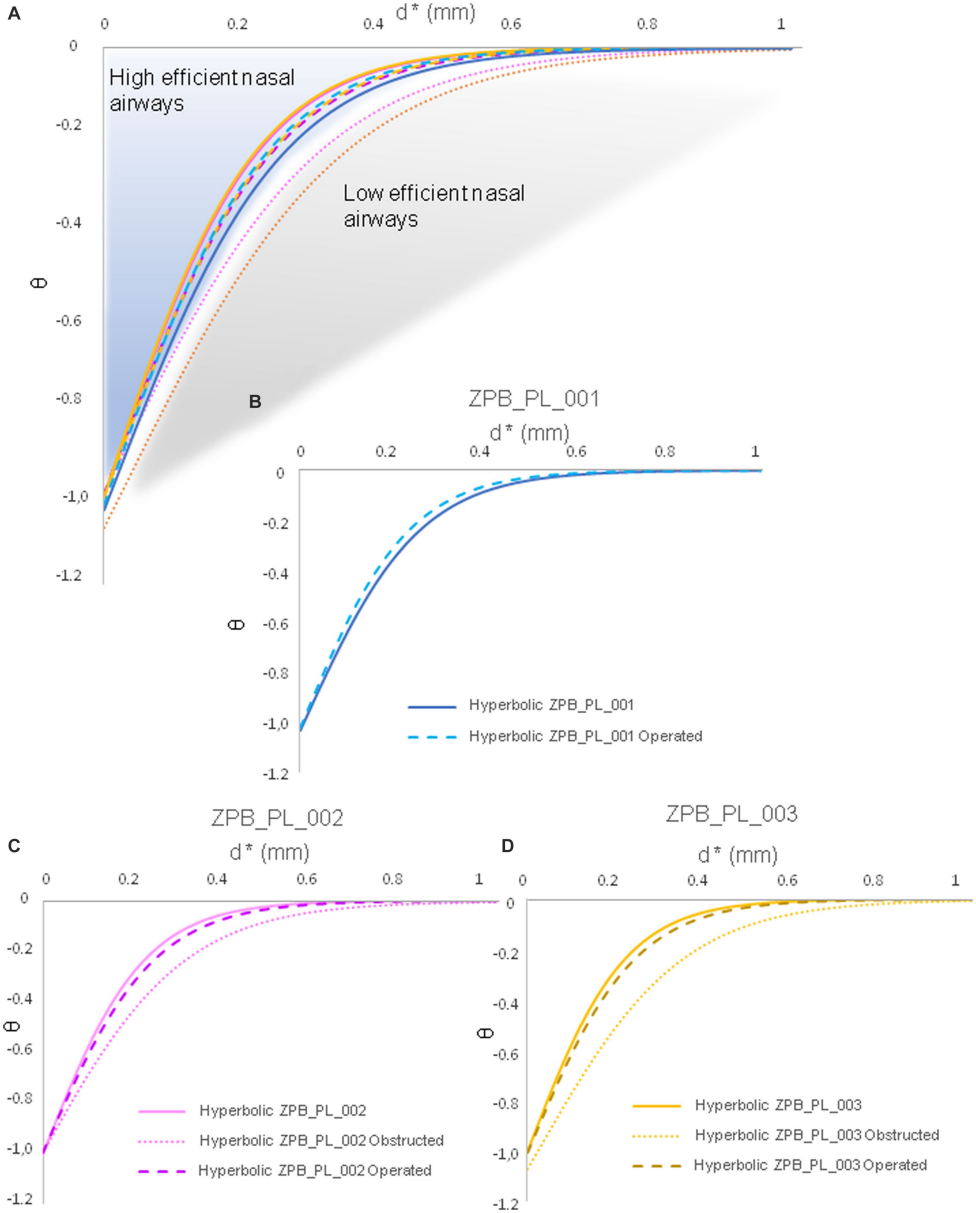
Humidity data collapsed into a hyperbolic function. These hyperbolic functions are generated based on the functions used by Burgos, 2014 (32). The average humidity data are from Tables 1–8. These hyperbolic functions describe the acclimation function specific to each nasal airway of each specimen. **(A)** General cases of all specimens. **(B)** Functional hyperbola of specimen ZPB_PL_001. **(C)** Functional hyperbola of specimen ZPB_PL_002. **(D)** Functional hyperbola of specimen ZPB_PL_003. Case 01 (ZPB_PL_001), Case 02 (ZPB_PL_002), and Case 03 (ZPB_PL_003) are shown.

This principle is highly dependent on the airflow topology. Therefore, the average data on the temperature and absolute humidity variables were collapsed to fit a hyperbolic function, generating a unique hyperbolic curve and providing a robust signal for the nasal airway (Figures 5, 6). In Figures 5, 6, a loss of hyperbolic function was observed when ZPB_PL_003 was obstructed (Figures 3B, 4). This indicated that the obstruction induced in Cases 03 and 02 negatively affected the resolution of the flow during acclimatization. Our results confirm that Flowgy has the analytical ability to resolve and detect small nasal obstructions. We developed a method to validate the efficiency of this function using hyperbolic curves and the obtained data, (32). A mesh convergence test was performed using the specimen from Case 01 [similar to Burgos (32)] to test the independence of the results with respect to mesh size. Three mesh resolutions were applied to this specimen: coarse (approximately 1.5 million elements), medium (approximately 3.5 million elements), and fine (approximately 7 million elements, approximately half of those used in this study). Pressure and velocity measurements were obtained at common locations (right and left nostrils and choana) and compared between mesh sizes (see Table 10). The relative error (RE) was calculated using the following formula:


$$RE=|\frac{\mathrm{Value finest mesh}-\mathrm{Value coarse mesh}}{\mathrm{value finest mesh}}|x100\%$$

In all cases, the relative error decreased from 50 to 78% when moving from a low mesh size to a medium mesh size. In all cases, when moving to a fine mesh size (high density of elements), the flow field was accurately represented, achieving convergence for these variables (temperature, humidity, pressure, and velocity; Table 10).

### Statistical analysis

In this study, the Wilcoxon test was used to quantify the effects of surgery. The Wilcoxon test (a non-parametric test) for related samples (also known as the Wilcoxon signed-rank test) could be a suitable option for comparing measurements in the same case before and after the operation, especially if the sample size is small (no more than 10 samples). The data on which the statistical analyses were performed are the mean data (θ) for both temperature and humidity obtained by applying Burgos (32). The data in Supplementary Table S1; Figure S1 indicate that the unobstructed operation in Case 01 had a statistically significant loss on the normal function of acclimatizing air in the nasopharynx. In the discussion on functional hyperbolics. The data from Case 01 post-operation aligned with that of other cases post-operation, suggesting a restoration of nasopharyngeal function and successful adaptation to air acclimatization. Based on an experiment with replicates to test the significance of the effect of the operation on these large felids, we started by testing the correlation between the genetically related data of Cases 02 and 03. Using the ordinary least squares (OLS) method with linear regression, the regression slope was almost 1 (slope = 0.99) with a Pearson correlation coefficient of *r* = 0.99. This indicated that the variables behaved similarly and supported the fact that they are two sibling specimens or even twins (Supplementary Table S2; Figure S2A). The operation in Cases 02 and 03 consisted of eliminating the induced obstruction and replicating the original obstruction from Case 01, both in size and anatomical position (Table 9). The statistical data for the Wilcoxon test in both the original cases and the cases after the operation, collected in Supplementary Tables S3–S5, indicated no significant differences between the operation and original states. Supplementary Figure S2B indicates a linear regression with r = 0.99 between Cases 02 and 03 after the operation. Supplementary Figures S4, S5 show very similar violin and box distributions between the operated and original cases. The operated case with the highest data dispersion was used.

### Conclusion

In conclusion, our results confirmed that Flowgy has the analytical ability to resolve and detect small nasal obstructions. Using the obtained data, we developed a method to validate the efficiency of this function using hyperbolic curves (32). The integration of engineering tools such as CFD and Flowgy in veterinary medicine represents a significant leap forward in our ability to understand and treat respiratory conditions in animals (3–6). These tools provide a non-invasive, detailed, and accurate analysis of airflow dynamics, significantly enhancing our ability to diagnose and manage these conditions (7–18). This improves the health and well-being of these animals and contributes to our broader understanding of their physiology and adaptations, thereby informing conservation efforts (1–6). As we continue to explore and develop these tools, we expect further advances in this exciting field.

## Discussion

This study demonstrated the power of virtual surgery and CFD virtual computation (33–36). The results based on small operations on the replicate lions have shown that the Flowgy software has the power and definition to detect these small obstructions and quantify them through the modeling resulting from the simulations. In the detection and virtual operation presented by Flowgy software, this tool will help visualize possible pathological diseases in large mammals, specifically those that are not easy to handle due to their aggressive and dangerous behavior, such as large felids.

Using sibling specimens (subjects with high genetic relatedness) provides a way to test the potential of virtual surgery and the analytical computing capabilities of Flowgy. Very minor operations or very small obstructions provide interesting results regarding the functional stability of airways (33, 34). As observed with the results from obstructions and virtual obstructive surgery in lions, small variations in the operation result in variations in the hyperbolic function. This indicated that Flowgy has the computational resolution to detect small disturbances in the topologies of the specimens and can precisely predict whether the alterations affect airway function compared with its normal hyperbolic function. Furthermore, in all instances, the hyperbolic functions of the treated cases overlapped with the normal hyperbolic function of the healthy cases before the obstructive intervention. This includes ZPB_PL_001 (Case 01), in which only the natural obstruction was treated. Although we do not have an initial healthy case for comparison with the new hyperbolic function of Case 01, it was in the same region as the other healthy and treated hyperbolic functions of Cases 02 and 03. In addition, the hyperbolic function of ZPB_PL_001 has a larger slope and is above the base hyperbola. This indicates that the operation improved the respiratory function by more than 100%.

This study demonstrates the power of the Flowgy program for detecting small anomalies in the nasal airways and computing complex nasal topologies, such as those of great felids such as *Panthera leo* (3–6). Additionally, it shows that Flowgy is a highly effective tool for routinely diagnosing and preventing respiratory diseases. This will help us to achieve control over respiratory diseases, in the preventive diagnosis for veterinary specialists, and in the conservation of large mammals in zoos and extensive natural reserves.

## Data availability statement

The original contributions presented in the study are included in the article/Supplementary material, further inquiries can be directed to the corresponding author.

## Ethics statement

Ethical review and approval were not required for the study on animals in accordance with the local legislation and institutional requirements. Written informed consent was obtained from the owners for the participation of their animals in this study.

## Author contributions

AP-R and MBu conceived and designed the study. BM scanned the specimens. AP-R and MBu analyzed the data. AP-R and MBu wrote the paper with the input of FE, DS-P, and MBa. All authors contributed to the article and approved the submitted version.

## Footnotes

^1^https://www.flowgy.com/

^2^https://www.openfoam.com/
