# Supplementary material for: Advancements in veterinary medicine: the use of Flowgy for nasal airflow simulation and surgical predictions in big felids (a case study in lions)
Source: Front Vet Sci. 2024 Jan 24;10:1181036. doi: 10.3389/fvets.2023.1181036 (PMC10847520; doi:10.3389/fvets.2023.1181036)

Supplementary Material

Advancements in Veterinary Medicine: The Use of Flowgy for Nasal Airflow Simulation and Surgical Predictions in big Felids (a case of study in lions).

Manuel Burgos [1], Alejandro Pérez-Ramos [2*], Baptiste Mulot[3]; Daniel Sanz-Prieto[4], Francisco Esteban[5]; Markus Bastir [4]

[1]Dpto. de Ingeniería Térmica y de Fluidos, Universidad Politécnica de Cartagena, Murcia, Spain

[2]Dpto. de Ecología y Geología, Facultad de Ciencias, Universidad de Málaga, 29071, Málaga, Spain

[3]ZooParc de Beauval & Beauval Nature, 41110 Saint-Aignan, France.

[4]Department of Paleobiology, Museo Nacional de Ciencias Naturales, 28006 Madrid, Spain.

[5]Servicio Andaluz de Salud, Sevilla, Spain. Hospital Universitario Virgen del Rocío, Servicio de Otorrinolaringología.

*** Correspondence:**
Corresponding Author
pera@uma.es

1 Supplementary Data

Table 1. Data of the specimen of *Panthera leo* ZPB_PL_001. Average data of temperature (Temp.) in Kelvin units and absolute humidity (Humid.) in Kg/m^3 units variables in the three simulated scenarios, at 28 ºC or 301.15 K (temperate biome) with a RH of 60%; at 40 ºC or 313.15 K (desert biome) with a RH of 05%; and at 5 ºC or 278.18 K (arctic or high mountain biome) with a RH of 10%. In addition, there is a standardization of the cut coordinates (distance) so that they are homologous and equidistant by subtracting the first coordinate value a from all the coordinate values.

Study Slices	xCoord	Distance	Temp. 28	Temp. 40	Temp. 5	Humid. 60	Humid. 05	Humid. 10
1	0.6858	0.0000	301.1500	313.1500	278.1500	0.0163	0.0026	0.0007
2	0.6478	0.0380	306.3630	312.4040	294.7070	0.0312	0.0241	0.0231
3	0.6232	0.0626	310.6410	311.7930	308.3750	0.0440	0.0425	0.0422
4	0.5986	0.0872	311.4470	311.6790	310.9920	0.0466	0.0463	0.0462
5	0.5739	0.1118	311.6320	311.6530	311.5930	0.0473	0.0472	0.0472
6	0.5493	0.1365	311.6480	311.6500	311.6450	0.0473	0.0473	0.0473
7	0.5247	0.1611	311.6500	311.6500	311.6490	0.0473	0.0473	0.0473
8	0.5001	0.1857	311.6500	311.6500	311.6480	0.0473	0.0473	0.0473
9	0.4755	0.2103	311.5100	311.6700	311.2170	0.0469	0.0467	0.0467

Table 2. Data from the operated *Panthera leo* specimen ZPB_PL_001. Average data of temperature (Temp.) in Kelvin units and absolute humidity (Humid.) in Kg/m^3 units variables in the three simulated scenarios, at 28 ºC or 301.15 K (temperate biome) with a RH of 60%; at 40 ºC or 313.15 K (desert biome) with a RH of 05%; and at 5 ºC or 278.18 K (arctic or high mountain biome) with a RH of 10%. In addition, there is a standardization of the cut coordinates (distance) that they are homologous and equidistant by subtracting the first coordinate value a from all the coordinate values.

Study Slices	xCoord	Distance	Temp. 28	Temp. 40	Temp. 5	Humid. 60	Humid. 05	Humid. 10
1	0.6858	0.0000	301.1500	313.1500	278.1500	0.0163	0.0026	0.0007
2	0.6478	0.0380	307.0950	312.2990	297.0560	0.0334	0.0272	0.0263
3	0.6232	0.0626	310.9480	311.7490	309.3700	0.0450	0.0439	0.0437
4	0.5986	0.0872	311.5220	311.6680	311.2320	0.0469	0.0467	0.0466
5	0.5739	0.1118	311.6380	311.6520	311.6120	0.0473	0.0473	0.0473
6	0.5493	0.1365	311.6490	311.6500	311.6480	0.0474	0.0474	0.0474
7	0.5247	0.1611	311.6500	311.6500	311.6490	0.0474	0.0474	0.0474
8	0.5001	0.1857	311.6500	311.6500	311.6490	0.0474	0.0474	0.0474
9	0.4755	0.2103	311.4910	311.6730	311.1570	0.0469	0.0467	0.0466

Table 3. Data of the specimen of *Panthera leo* ZPB_PL_003. Average data of temperature (Temp.) in Kelvin units and absolute humidity (Humid.) in Kg/m^3 units variables in the three simulated scenarios, at 28 ºC or 301.15 K (temperate biome) with a RH of 60%; at 40 ºC or 313.15 K (desert biome) with a RH of 05%; and at 5 ºC or 278.18 K (arctic or high mountain biome) with a RH of 10%. In addition, there is a standardization of the cut coordinates (distance) that they are homologous and equidistant by subtracting the first coordinate value a from all the coordinate values.

Study Slices	xCoord	Distance	Temp. 28	Temp. 40	Temp. 5	Humid. 60	Humid. 05	Humid. 10
1	0.8733	0.0000	301.1510	313.1520	278.1490	0.0163	0.0026	0.0007
2	0.7836	0.0897	309.0290	312.0210	303.1560	0.0390	0.0354	0.0347
3	0.7367	0.1366	310.8260	311.7660	308.9570	0.0445	0.0433	0.0431
4	0.6897	0.1836	311.4400	311.6800	310.9630	0.0466	0.0462	0.0462
5	0.6427	0.2306	311.5990	311.6570	311.4910	0.0472	0.0471	0.0471
6	0.5958	0.2775	311.6170	311.6550	311.5480	0.0472	0.0472	0.0472
7	0.5488	0.3245	311.6140	311.6550	311.5380	0.0472	0.0472	0.0472
8	0.5018	0.3715	311.5810	311.6600	311.4370	0.0472	0.0471	0.0470
9	0.4548	0.4184	311.4700	311.6760	311.0930	0.0468	0.0466	0.0466

Table 4. Data from the obstructed *Panthera leo* specimen ZPB_PL_003. Average data of temperature (Temp.) and relative humidity (Humid. or RH) variables in the three simulated scenarios, at 28 ºC or 301.15 K (temperate biome) with a RH of 60%; at 40 ºC or 313.15 K (desert biome) with a RH of 05%; and at 5 ºC or 278.18 K (arctic or high mountain biome) with a RH of 10%. In addition, there is a standardization of the cut coordinates (distance) that they are homologous and equidistant by subtracting the first coordinate value a from all the coordinate values.

Study Slices	xCoord	Distance	Temp. 28	Temp. 40	Temp. 5	Humid. 60	Humid. 05	Humid. 10
1	0.8733	0.0000	301.1510	313.1520	278.1490	0.0163	0.0026	0.0007
2	0.7836	0.0897	304.5810	312.6590	289.0770	0.0261	0.0167	0.0155
3	0.7367	0.1366	309.7760	311.9150	305.5580	0.0413	0.0387	0.0382
4	0.6897	0.1836	311.1960	311.7140	310.1430	0.0458	0.0451	0.0449
5	0.6427	0.2306	311.5910	311.6580	311.4610	0.0472	0.0471	0.0471
6	0.5958	0.2775	311.6160	311.6550	311.5450	0.0473	0.0472	0.0472
7	0.5488	0.3245	311.6140	311.6550	311.5390	0.0473	0.0472	0.0472
8	0.5018	0.3715	311.5810	311.6600	311.4370	0.0472	0.0471	0.0471
9	0.4548	0.4184	311.4700	311.6760	311.0930	0.0469	0.0467	0.0466

Table 5. Data from the operated *Panthera leo* specimen ZPB_PL_003. Average data of temperature (Temp.) in Kelvin units and absolute humidity (Humid.) in Kg/m^3 units variables in the three simulated scenarios, at 28 ºC or 301.15 K (temperate biome) with a RH of 60%; at 40 ºC or 313.15 K (desert biome) with a RH of 05%; and at 5 ºC or 278.18 K (arctic or high mountain biome) with a RH of 10%. In addition, there is a standardization of the cut coordinates (distance) that they are homologous and equidistant by subtracting the first coordinate value a from all the coordinate values.

Study Slices	xCoord	Distance	Temp. 28	Temp. 40	Temp. 5	Humid. 60	Humid. 05	Humid. 10
1	0.8733	0.0000	301.1510	313.1520	278.1490	0.0163	0.0026	0.0007
2	0.7836	0.0897	308.3400	312.1200	300.9660	0.0370	0.0325	0.0317
3	0.7367	0.1366	310.7160	311.7820	308.6030	0.0442	0.0428	0.0426
4	0.6897	0.1836	311.4150	311.6830	310.8780	0.0465	0.0461	0.0461
5	0.6427	0.2306	311.5990	311.6570	311.4890	0.0472	0.0471	0.0471
6	0.5958	0.2775	311.6140	311.6550	311.5380	0.0472	0.0472	0.0472
7	0.5488	0.3245	311.6120	311.6550	311.5320	0.0473	0.0472	0.0472
8	0.5018	0.3715	311.5800	311.6600	311.4330	0.0472	0.0471	0.0471
9	0.4548	0.4184	311.4670	311.6770	311.0810	0.0469	0.0466	0.0466

Table 6. Data of the specimen of *Panthera leo* ZPB_PL_002. Average data of temperature (Temp.) in Kelvin units and absolute humidity (Humid.) in Kg/m^3 units variables in the three simulated scenarios, at 28 ºC or 301.15 K (temperate biome) with a RH of 60%; at 40 ºC or 313.15 K (desert biome) with a RH of 05%; and at 5 ºC or 278.18 K (arctic or high mountain biome) with a RH of 10%. In addition, there is a standardization of the cut coordinates (distance) so that they are homologous and equidistant by subtracting the first coordinate value a from all the coordinate values.

Study Slices	xCoord	Distance	Temp. 28	Temp. 40	Temp. 5	Humid. 60	Humid. 05	Humid. 10
1	0.8733	0.0000	301.1500	313.1500	278.1500	0.0163	0.0026	0.0007
2	0.7836	0.0897	308.8670	312.0450	302.6470	0.0385	0.0346	0.0340
3	0.7367	0.1366	310.8770	311.7590	309.1210	0.0447	0.0436	0.0434
4	0.6897	0.1836	311.4680	311.6760	311.0500	0.0467	0.0464	0.0463

5	0.6427	0.2306	311.6170	311.6550	311.5470	0.0472	0.0472	0.0472
6	0.5958	0.2775	311.5100	311.6700	311.2170	0.0469	0.0467	0.0467
7	0.5488	0.3245	311.6420	311.6510	311.6250	0.0473	0.0473	0.0473
8	0.5018	0.3715	311.6010	311.6570	311.4990	0.0472	0.0472	0.0472
9	0.4548	0.4184	311.4350	311.6810	310.9860	0.0468	0.0465	0.0465

Table 7. Data from the obstructed *Panthera leo* specimen ZPB_PL_002. Average data of temperature (Temp.) in Kelvin units and absolute humidity (Humid.) in Kg/m^3 units variables in the three simulated scenarios, at 28 ºC or 301.15 K (temperate biome) with a RH of 60%; at 40 ºC or 313.15 K (desert biome) with a RH of 05%; and at 5 ºC or 278.18 K (arctic or high mountain biome) with a RH of 10%. In addition, there is a standardization of the cut coordinates (distance) so that they are homologous and equidistant by subtracting the first coordinate value a from all the coordinate values.

Study Slices	xCoord	Distance	Temp. 28	Temp. 40	Temp. 5	Humid. 60	Humid. 05	Humid. 10
1	0.8733	0.0000	301.1490	313.1500	278.1500	0.0163	0.0026	0.0007
2	0.7836	0.0897	304.4240	312.6830	288.5680	0.0386	0.0159	0.0147
3	0.7367	0.1366	309.7990	311.9080	305.7220	0.0447	0.0390	0.0385
4	0.6897	0.1836	311.2200	311.7100	310.2300	0.0467	0.0452	0.0450
5	0.6427	0.2306	311.6100	311.6560	311.5170	0.0473	0.0472	0.0472
6	0.5958	0.2775	311.5020	311.6700	311.2140	0.0469	0.0468	0.0467
7	0.5488	0.3245	311.6420	311.6510	311.6260	0.0473	0.0473	0.0473
8	0.5018	0.3715	311.6010	311.6570	311.4990	0.0472	0.0472	0.0472
9	0.4548	0.4184	311.4300	311.6810	310.9860	0.0468	0.0465	0.0465

Table 8. Data from the operaded *Panthera leo* specimen ZPB_PL_002. Average data of temperature (Temp.) in Kelvin units and absolute humidity (Humid.) in Kg/m^3 units variables in the three simulated scenarios, at 28 ºCor 301.15 K (temperate biome) with a RH of 60%; at 40 ºCor 313.15 K (desert biome) with a RH of 05%; and at 5 ºCor 278.18 K (arctic or high mountain biome) with a RH of 10%. In addition, there is a standardization of the cut coordinates (distance) so that they are homologous and equidistant by subtracting the first coordinate value a from all the coordinate values.

Study Slices	xCoord	Distance	Temp. 28	Temp. 40	Temp. 5	Humid. 60	Humid. 05	Humid. 10
1	0.8733	0.0000	301.1500	313.1500	278.1500	0.0163	0.0026	0.0007
2	0.7836	0.0897	308.1780	312.1440	300.4570	0.0365	0.0317	0.0310
3	0.7367	0.1366	310.7670	311.7750	308.7670	0.0444	0.0431	0.0429
4	0.6897	0.1836	311.4430	311.6790	310.9650	0.0466	0.0463	0.0462
5	0.6427	0.2306	311.6170	311.6550	311.5450	0.0473	0.0472	0.0472
6	0.5958	0.2775	311.5070	311.6700	311.2070	0.0469	0.0467	0.0467
7	0.5488	0.3245	311.6400	311.6510	311.6190	0.0473	0.0473	0.0473
8	0.5018	0.3715	311.6000	311.6570	311.4950	0.0472	0.0472	0.0472

9	0.4548	0.4184	311.4320	311.6820	310.9740	0.0468	0.0465	0.0465

2. Statistical analysis

Table 1S. Wilcoxon test for related samples, Case 01 original (within obstruction) Vs Case 01 Operated (Without obstruction). The significance level exceeds (α: 0,05), so the differences between the original and the operated obstructed situation are statistically significant with a p-value: 0.186, using the Monte Carlo bootstrapping method for small samples.

Case 01: Original-Operated
N: 9
Mean: -0,18166 Mean: -0,16996
Median: -0,0132 Median: -0,0122
t test
Mean difference: 0,0117 95% conf.: (-0,0067081 0,030108)
t : -1,4657 p (same mean): 0,18091
Exact: p (same mean): 0,125
Sign test
r: 4 p (same median): 0,375
Wilcoxon test:
W: 13
Normal appr. z : 1,4832 p (same median): 0,13801
Monte Carlo (n=99999): p (same median): 0,18603
Exact: p (same median): 0,1875

Figure 1S. Violin and box plot between Case 01 Original and Case 01 Operated. On the Cartesian Y axis, standardization of temperature and humidity data (θ) obtained from the 3 simulations (28ºC at 60%; 40ºC at 05%; 05ºC at 10%) applying the method described in the work of Burgos, 2014.

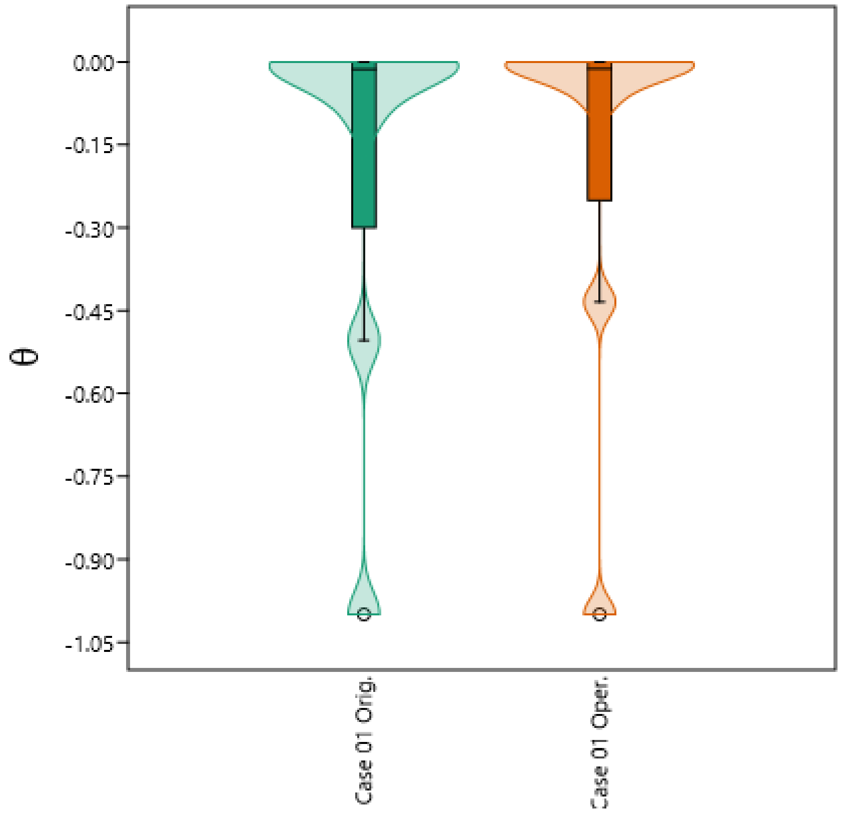

Table 2S. OLS regression between Case 2 and Case 3. Linear regression applied to the average data (θ) for both temperature and humidity obtained by applying Burgos, 2014. A). Original Case in both Case 2 and Case 3. The statistical data indicate a slope of 0.99 and a Pearson correlation coefficient r=0.99. (See Figure 2SA).

Ordinary Least Squares Regression: Case 02-Case 03 Orig.
Slope a: 0,99818 Std. error a: 0,0078371
t: 127,37 p (slope): 4,8507E-13
Intercept b: 0,0014061 Std. error b: 0,002711
95% bootstrapped confidence intervals (N=1999):
Slope a: (0,93331, 1,1717)
Intercept b: (-0,0033393, 0,0063274)
Correlation:
r: 0,99978
r2: 0,99957
t: 127,37
p (uncorr.): 4,8507E-13
Permutation p: 0,0001

Table 3S. OLS regression between Case 2 and Case 3. Linear regression applied to the average data (θ) for both temperature and humidity obtained by applying Burgos, 2014. B). Operated Case in both Case 2 and Case 3. The statistical data indicate a slope of 0.99 and a Pearson correlation coefficient r=0.99. (See Figure 2SB).

Ordinary Least Squares Regression: Case 02 -Case 03 Oper.
Slope a: 0,99724 Std. error a: 0,007756
t: 128,58 p (slope): 4,5401E-13
Intercept b: 0,0012463 Std. error b: 0,0027336
95% bootstrapped confidence intervals (N=1999):
Slope a: (0,92551, 1,0517)
Intercept b: (-0,003588, 0,0058646)
Correlation:
r: 0,99979
r2: 0,99958
t: 128,58
p (uncorr.): 4,5401E-13
Permutation p: 0,0001

Figure 2S. OLS regression between Case 2 and Case 3 plots. Linear regression applied to the average data (θ) for both temperature and humidity obtained by applying Burgos, 2014. A). Original Case in both Case 2 and Case 3. The statistical data indicate a slope of 0.99 and a correlation of 0.999 (both r and r^2), see Table 2S; B). Operated Case in both Case 2 and Case 3. The statistical data indicate a slope of 0.99 and a correlation of 0.999 (both r and r^2), see Table 3S.

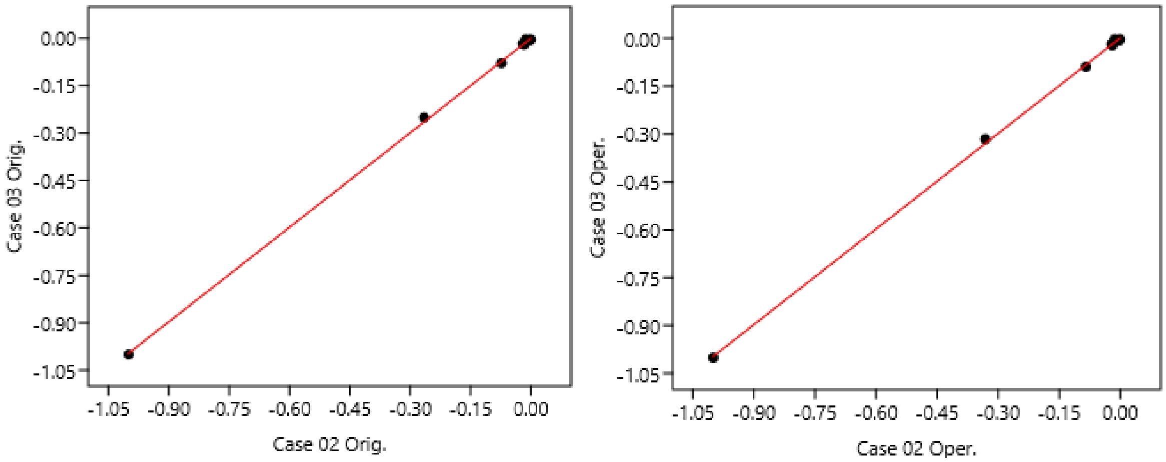

Figure 3S. Violin and box plots comparing Case 02 and 03. It has a very similar data dispersion and based on the statistical data of the linear regression applying OLS, a higher similarity is observed.

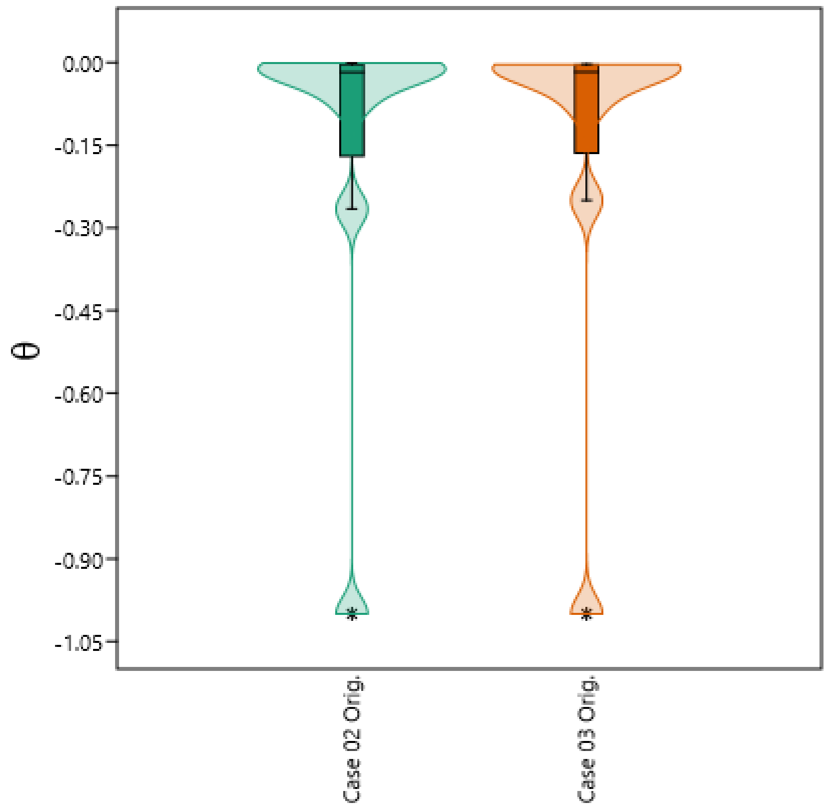

Table 4S. Wilcoxon test for Case 02 original Vs Case 02 Operated (Without obstruction). The significance level is less than (α: 0,05), so the differences between the original and the operated situation are not statistically significant with a p-value of 0.015, using the Monte Carlo bootstrapping method for small samples. This indicates that the operation has been close to 100% successful.

Case 02 Original-Operated
N: 9
Mean: -0,15546 Mean: -0,16429
Median: -0,0175 Median: -0,0198
t test
Mean difference: 0,0088333 95% conf.: (-0,0077691 0,025436)
t : 1,2269 p (same mean): 0,25474
Exact: p (same mean): 0
Wilcoxon test:
W: 28
Normal appr. z: 2,3664 p (same median): 0,01796
Monte Carlo (n=99999): p (same median): 0,01567
Exact: p (same median): 0,015625

Figure 4S. Violin and box plots comparing Cases 02: original, obstructed and operated. The obstructed Case 02 has a higher data dispersion due to the correct functional loss of airflow acclimatization. The operated Case 02 has a very similar data dispersion to the original Case 02, based on Table 4S, they are statistically equal. The operation corrects almost 100% of the millimeter scale induced obstruction.

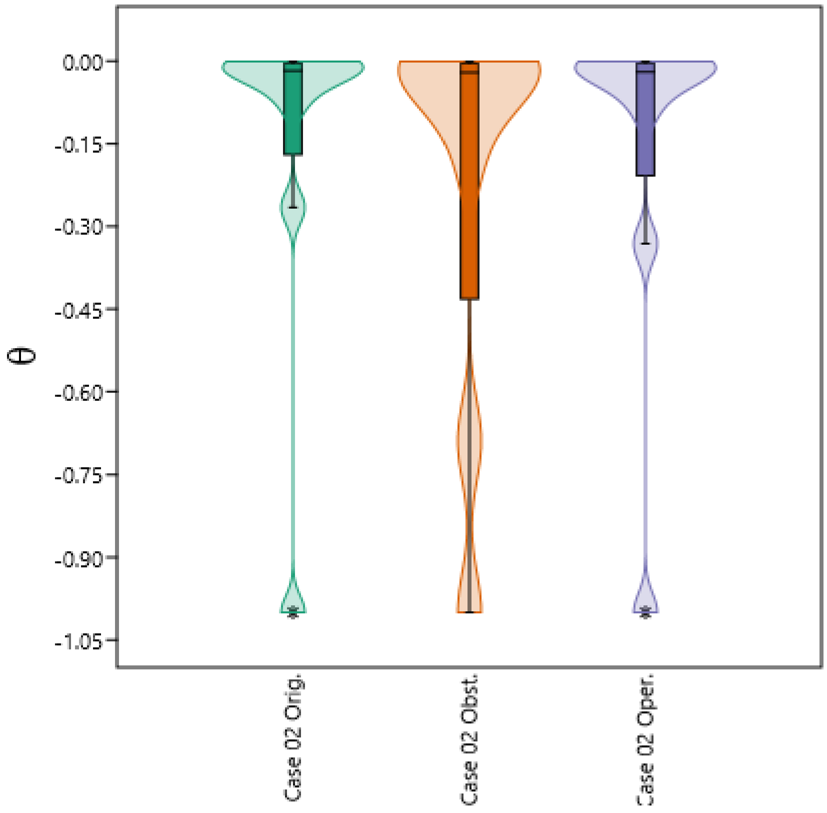

Table 5S. Wilcoxon test for Case 03 original Vs Case 03 Operated (Without obstruction). The significance level is less than (α: 0,05), so the differences between the original and the operated situation are not statistically significant with a p-value of 0.015, using the Monte Carlo bootstrapping method for small samples. This indicates that the operation has been close to 100% successful.

Case 03 Original-Operated
N: 9
Mean: -0,15377 Mean: -0,16259
Median: -0,017 Median: -0,0175
t test
Mean difference: 0,0088222 95% conf.: (-0,0077549 0,025399)
t: 1,2272 p (same mean): 0,25462
Exact: p (same mean): 0

Wilcoxon test:
W: 28
Normal appr. z: 2,3707 p (same median): 0,017756
Monte Carlo (n=99999): p (same median): 0,01522
Exact: p (same median): 0,015625

Figure 5S. Violin and box plots comparing Case 03: original, obstructed and operated. The obstructed Case 03 has a higher data dispersion due to the correct functional loss of airflow acclimatization. The operated Case 03 has a very similar data dispersion to the original Case 03, based on Table 5S, they are statistically equal. The operation corrects almost 100% of the millimeter scale induced obstruction.

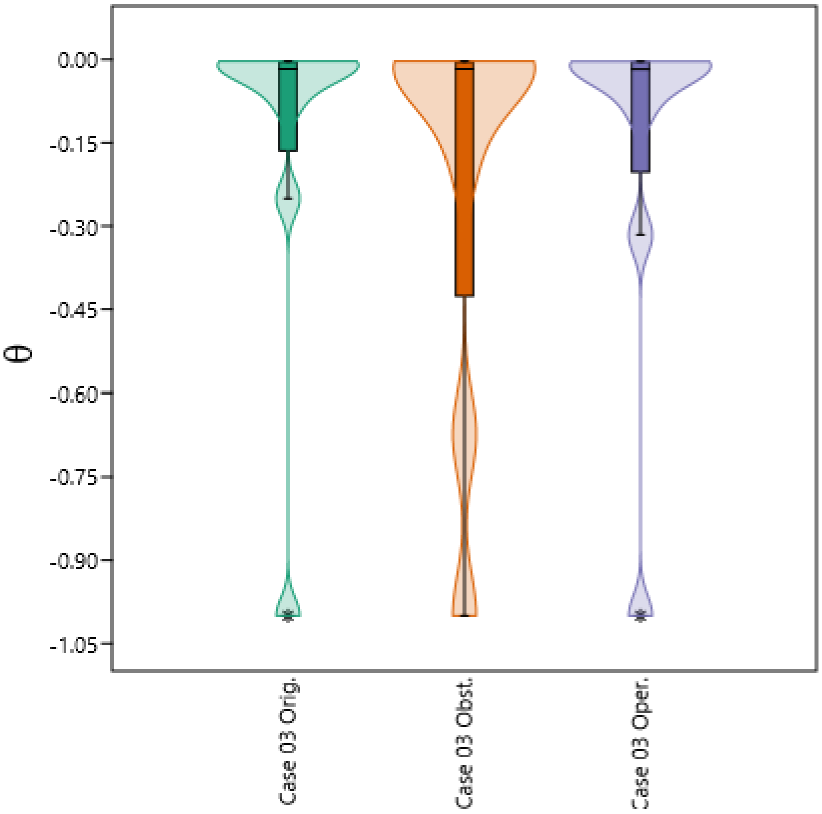

Supplement: Supplementary file 1 [file Data_Sheet_1.PDF]
